# Supplementary material for: Bootstrapping and Pinning down the Root Meristem; the Auxin–PLT–ARR Network Unites Robustness and Sensitivity in Meristem Growth Control
Source: Int J Mol Sci. 2021 Apr 29;22(9):4731. doi: 10.3390/ijms22094731 (PMC8125115; doi:10.3390/ijms22094731)
Supplement: Supplementary file 1 [file ijms-22-04731-s001.zip › ijms-1166354-supplementary.pdf]

Material and methods (extended supplement version)

In this paper we use 3 distinct but related models to better understand how the feedbacks between Auxin, PLETHORA (PLT) and the ARR-Bs control Arabidopsis root tip growth dynamics. The first model is a 2D anatomically detailed root tip model based on the Salvi et al. 2020 model with some minor alterations made to support additional experiments. The second model is a single compartment ODE system that captures in a simplified manner the essence of the Auxin-PLT positive feedback loop dynamics. The final model is an extension of this second model into 2 separate but connected compartments incorporating polar auxin transport between these compartments.

Root tip model

The model recently published in Salvi et al., 2020 serves as the basis for the root tip model used in the current paper. Minor alterations in model settings and parameter values were made based on new insights (see Model alterations relative to Salvi et al., 2020). The parameters described in Supplementary tables 1 to 11 are the resulting default parameters, used in standard simulations corresponding to *in planta* conditions. In the section *Variations in model parameter settings* we discuss modifications in model parameter settings used to investigate impact of various aspects of root tip auxin, PLT and growth dynamics on meristem size control.

In order to facilitate the understanding of the current paper independently of the Salvi et al., 2020 paper below we describe the building blocks of the root tip model.

Tissue architecture

The root tip model incorporates an idealized, wedge shape root tip anatomy, with built in intracellular regulatory dynamics, intercellular hormone and protein transport, and cell growth, division, expansion and differentiation. The model describes all tissue types present in the adult Arabidopsis root, from inside to outside these are the vasculature, pericycle, endodermis, cortex, epidermis, and in the meristem zone the lateral root cap. For the root tip we also incorporate the quiescent center, stem cells, columella cells and root cap (Figure 1A). Longitudinally, the intracellular regulatory dynamics determine developmental stages of the cell, dividing the root into separate developmental zones (Figure 1A). The model is grid based, meaning that individual cells, but also cell walls occupy a set of connected grid points. Modeled cell sizes and shapes are based on experimental data, and cell widths are dependent on tissue type (18 micron epidermis, 20 micron cortex, 12 micron endodermis, 8 micron pericycle and 6 micron vasculature). Maximum cell height depends on the developmental

phase of the cell (just divided cells have a height of 8 micron and increase to 16 micron before dividing, and maximally elongated cells reach a height of 144 micron).

To simulate realistic auxin patterning, we incorporate cell type specific and zone specific expression patterns for both the auxin exporting PIN proteins and the auxin importing AUX/LAX proteins (Figure 3A), as done in previous modeling studies (Band et al., 2014; van den Berg et al., 2016; Grieneisen et al., 2007; Mähönen et al., 2014) and identical to Salvi et al., 2020.

One source of auxin is influx from the shoot, which is modeled via an extracellular auxin level on top of the higher vascular cell. The second source of auxin is local root tip production, which plays an important role in root auxin patterning (Ljung et al., 2005; Stepanova et al., 2008). Next to a baseline auxin production occurring in all cells, we therefore included elevated auxin production in the QC, columella and lateral root cap (auxin production in the QC 50x basal rate, SCN 25x basal rate, and LRC 10x basal rate).

Growth dynamics

We use the same simplified algorithm for cellular growth, division and expansion dynamics here that we used in Salvi et al., 2020 and was originally developed in Mahonen 2014, which limits growth dynamics to strictly rectangular cells above the tip, and ignoring cell division in the columella, lowest lateral root cap cells and in the QC. Those cells that do grow increase in size through the addition of a cell-width sized row of grid points to their shootward end, and by the upwards shifting of above lying cells as a consequence of this increase in volume. To maintain symplastic growth and thus avoid unrealistic sliding of cells past one another in our simulations, we assumed that cell growth and developmental transitions are coordinated across neighboring cells (see section on radial coordination).

Cellular growth events lead to the increase of cytoplasmic volume only in case of meristematic cell growth, but not as a result of vacuolar expansion driven cell expansion. In meristematic growth cytoplasmic concentrations are therefore diluted proportionally to cytosolic expansion. Once cells have doubled their size (change in height from 8 to 16 micron) they divide. The resulting daughter cells inherit the PIN and AUX/LAX patterns of the maternal cells. When a cell enters a new developmental zone in which these patterns are different, the patterning of this cell is changed accordingly.

Zonation

Experimental data indicate that two separate zones can be distinguished inside the meristem. A first most proximal zone consists of actively dividing cells, whereas a second more distal zone that may comprise up to one third of the meristem consists of cells that hardly undergo division (Boudonck et al., 1998; Scacchi et al., 2010; Verbelen et al., 2006), Rather than undergoing rapid vacuolar expansion these cells still appear to be slowly growing cytoplasmatically. We therefore incorporated two distinct meristematic domains, simplifying matters by assuming no further divisions in the distal meristem zone.

Based on the observation that longitudinal root zonation is accompanied by a graded decline of division associated transcription factors and an increase of differentiation associated transcription factors (Wendrich et al., 2017), we incorporated into our model a generalized division transcription factor (DivTF), and elongation factor (ElongTF). If the division factor exceeds a certain threshold level cells are assumed to be meristematic, whereas if the elongation factor exceeds a certain threshold level cells are assumed to start elongation and differentiation. In case both the division and elongation factors are below their respective threshold levels cells are assumed to slowly grow cytoplasmically yet no longer divide (Figure 3D).

Following Mähonen et al, where we demonstrated that PLT overexpression maintains cell division, while PLT knockout results in meristem differentiation (Mähonen et al. 2014), we include PLT-dependent expression of DivTF and PLT-driven repression of ElongTF. Additionally, based on the role of ARR-mediated CK signalling in promoting cell elongation and differentiation (Pacifici et al., 2018; Takatsuka et al., 2018), we model ARR1 and ARR12 dependent induction of ElongTF. As elongation and differentiation also occur in full cytokinin signalling mutants, albeit delayed, we also model an ARR independent baseline production of ElongTF. Furthermore, the model includes the repressive effect of ARR1, mediated via KRP2, on DivTF.. Finally, we model a repression of ElongTF by divTF, which can be interpreted as the repressive effects of epigenetic modifications associated with stemness and division on cell elongation (Krichevsky et al., 2009). Combined this results in the following equations:

$$\frac{d\mathrm{DivTF}}{dt} = \frac{p_{Div} * Plts^2}{Plts^2 + KM_{Plts,Div}} * ((1 - ARRDivFrac) + \frac{ARRDivFrac * KM_{ARR1,Div}^2}{\mathrm{ARR1}^2 + \mathrm{KM}_{ARR1,Div}^2}) - d_{Div} * \mathrm{DivTF} \qquad (1)$$

, where p_{Div} is the maximum production rate of DivTF, $KM_{Plts,Div}$ is the PLT level for which the production is raised to half maximum, ARRDivFrac is the maximum fraction ARR1 induced KRP2 can repress DivTF production by, $KM_{ARR1,Div}$ is the ARR1 level for which the this repression is half maximum, and d_{Div} is the DivTF degradation rate.

$$\frac{d\text{ElongTF}}{dt} = p_{El} * \left(\frac{Divfrac * KM_{,El}^4}{\text{DivTF}^4\text{KM}_{,El}^4} + \frac{Pltsfrac * \text{KM}_{Plts,El}^2}{\text{Plts}^2 + KM_{Plts,El}^2} + \frac{ARRFrac * ARRs_{eff}^2}{ARRs_{eff}^2 + \text{KM}_{ARRs,El}^2}\right) - d_{El} * \text{ElongTF} \tag{2}$$
$$\text{ARRs}_{eff} = \text{ARR1} * ARR1maximumeffect + \text{ARR12}$$

, where p_{El} is the maximum production rate of ElongTF, Divfrac is the maximum fraction the DivTF can repress ElongTF production by, $KM_{Div,El}$ is the DivTF level for which this repression is half maximum, Pltsfrac is the maximum fraction PLT can repress p_{El} by, $KM_{Plts,El}$ is the PLT level for which this repression is half maximum, ARRfrac is the maximum fraction ARRs can raise ElongTF production by, $KM_{ARRs,El}$ is the ARR effect level at which said raise is half maximum, d_{El} is the degradation rate of ElongTF, and ARR1maximumeffect is the weight factor for ARR1 resulting in a stronger effect of ARR1 over ARR12 on ElongTF. Parameter values are given in Supplementary Table 1.

Sup Table 1: Growth parameters

Parameter	Explanation	Salvi2020 value	Current Value	Dimension
p_{Div}	Maximum production rate of division TF	0.01	0.01	$[]s^{-1}$
d_{Div}	Degradation rate of division TF	0.0001	0.0001	s^{-1}
$KM_{Plts,Div}$	*PLT concentration at which PLT induction of division TF is half maximum*	*40*	*35*	*[]*
ARRDivFrac	Maximum repression fraction of ARR1 on division	1.0	0.5	Dimensionless
$KM_{ARR1,Div}$	ARR1 level at which ARR repression of division TF is half maximum	40	40	[]
Divisionthreshold	*Division TF concentration above which cells divide rapidly*	*45*	*40*	*[]*
p_{El}	Maximum production rate of elongation TF	0.01	0.01	$[]s^{-1}$
d_{El}	Degradation rate of elongation TF	0.0001	0.0001	s^{-1}

DivElongFrac	Maximum fraction of elongation TF production that Division TF can block	0.5	0.5	Dimensionless
$KM_{Div,El}$	*Concentration of division TF at which it represses elongation TF to half its maximum capacity*	*30*	*25*	*[]*
PltsElongFrac	Maximum fraction of elongation TF production that PLT can block	0.25	0.25	Dimensionless
$KM_{Plts,El}$	Concentration of PLT at which it represses elongation TF to half its maximum capacity	20	20	[]
$KM_{ARRs,El}$	Combined ARR effect at which it induces elongation TF to half its maximum capacity	80	80	[]
Elongtreshold	Concentration of ElongTF at which cells start elongating	40	40	[]

Auxin signalling

We incorporate TIR1/AFB-AUX/IAA mediated transcriptional auxin signalling (Guilfoyle, 2015; Rouse et al., 1998) using the following equation:

$$\frac{dARF_{free}}{dt} = diss * (ARF_{total} - ARF_{free}) - assAR\,F_{free} * AUX/IAA$$
$$\frac{d\text{AUX/IAA}}{dt} = P_{AUX/IAA} - d_{AUX/IAA,basal} * AUX/IAA - d_{AUX/IAA,TIR} * auxin * AUX/IAA \quad (3)$$

, where diss is the rate of dissociation of the ARF-AUX/IAA complex, ass is the rate of ARF association with AUX/IAA, $P_{AUX/IAA}$ is the production rate of AUX/IAA, $d_{AUX/IAA,basal}$ is the basal degradation rate of AUX/IAA and $d_{AUX/IAA,TIR}$ is the TIR mediated degradation rate of AUX/IAA. where we assume a constant level of total ARF. Parameter values are provided in Supplementary Table 2.

Taking a QSSA for AUX/IAA and substituting this in the equation for ARF_free results in:

$$\frac{d\mathrm{ARF}_{free}}{dt} = diss * \left(ARF_{total} - ARF_{free}\right) - \frac{assARF_{free} * p_{AUXIAA}}{d_{AUX/IAA,basal} + d_{AUX/IAA,TIR1} * \mathrm{Auxin}} \quad (4)$$

Which can be rewritten as:

$$\frac{d\mathrm{ARF}_{free}}{dt} = diss * ARF_{total} - \left(diss + \frac{ass * P_{AUX/IAA}}{d_{AUX/IAA,basal} + d_{AUX/IAA<TIR1} * \mathrm{Auxin}}\right) * ARF_{free} \quad (5)$$

Based on recent data suggesting the importance of AUX/IAA dimerisation for auxin signalling (Farcot et al., 2015; Kim et al., 1997; Korasick et al., 2014), we subsequently rewrite the latter equation to obtain a second order relation between auxin levels and free ARF levels:

$$\frac{d\mathrm{ARF}_{free}}{dt} = diss * ARF_{total} - \left(diss + \frac{ass * P_{AUX/IAA}}{d_{AUXIAA,basal} + d^2_{AUX/IAA<TIR1} * \mathrm{Auxin}^2}\right) * ARF_{free} \quad (6)$$

Sup table 2: ARF AUX/IAA parameters

Parameter	Explanation	Previous value	Current value	dimension
Ass	Association ARF to AUXIAA	0.01	0.01	s^{-1}
Diss	Dissociation ARF from AUXIAA	0.001	0.001	s^{-1}
$P_{AUX/IAA}$	Multiplied with association	0.01	0.01	s^{-1}
dIbasal	Minimum division value association ARF	0.0002	0.0002	s^{-1}
DITIR1	*Determines auxin contribution disassociation*	*0.000800*	*0.000725*	$[]^{-1}s^{-.5}$
ARFtotal	Total ARF present in cell	100	100	[]

PLT transcription

Following Mähonen et al. we model the delayed induction of PLT transcritipion by high auxin levels (Mähönen et al., 2014). By incorporating an auxin signalling cascade of 4 transcription factors, with the fourth inducing PLT transcription we reproduce the experimentally observed delay. Additionally, we incorporate a repressive effect of ARR12 mediated CK signalling on PLT transcription. Combined this results in the following equations:

$$\frac{dA}{dt} = \frac{p_A * maxcorrection * ARF_{free}^2}{ARF_{free}^2 + KM_{ARF,A}^2} - d_A * A$$
$$\frac{dB}{dt} = \frac{p_B * maxcorrection * A^2}{A^2 + KM_{A,B}} - d_B * B$$
$$\frac{dC}{dt} = \frac{p_C * maxcorrection * B^2}{B^2 + KM_{B,C}^2} - d_C * C \quad (7)$$
$$\frac{dD}{dt} = \frac{p_D * maxcorrection * C^2}{C^2 + KM_{C,D}^2} - d_D * D$$

$$\frac{d\text{Plts}}{dt} = \frac{\frac{p_{Plts} * D^2}{D^2 + \text{KM}_{D,Plts}^2} * \text{KM}_{ARR12,Plts}^2}{ARR12^2 + \text{KM}_{ARR12,Plts}^2} - d_{Plts} * \text{Plts} \quad (8)$$

, where p_A,p_B,p_C,p_D. are the maximum production rates , $KM_{ARF,A}KM_{A,B}$, $KM_{B,C}$ $KM_{C,D}$, and $KM_{D,Plts}$ are the half-maximum saturation constants, and d_A,d_B,d_C,d_D,and d_{Plts} are the degradation rates of of A, B, C, D and PLT respectively. Parameter values are given in Supplementary Table 3.

Sup table 3: ABCD TF cascade parameters

Parameter	Description	Salvi2020 value	New value	dimension
$p_{A,B,C,D}$	Max production of ABCD TFs	0.0065	0.0065	[]*s^{-1}
d_{ABCD}	Decay rate of ABCD TFs	0.000065	0.000065	s^{-1}
$KM_{ARF,A}$	*Arf km for B production*	*55*	*60*	*[]*
$KM_{A,B}$	*A km for B production*	*55*	*60*	*[]*
$KM_{B,C}$	*B km for C production*	*55*	*60*	*[]*
$KM_{C,D}$	*C km for D production*	*55*	*60*	*[]*
Maxcorrection	*Corrects A-D for full activation capacity*	*1.3025*	*1.36*	*Dimensionless*

Additional PLT dynamics

PLT growth dilution

We incorporate cytoplasmic volume increase driven dilution of both cytoplasmic and TF genes, reasoning that as cells double their volume in order to divide so does the nucleus.

PLT movement

Following Mahonen et al. we incorporated the cell-to-cell movement of PLT proteins, with cell-to-cell transport rate dependent on cell type and location based on experimental data, as done in Salvi et al. 2020. For PLT parameters see Supplementary Table 4.

PLT removal in bottom columella cells

To compensate for the absence of columella and root cap cells growth, division and subsequent sloughing off in our model, which would result in dilution of PLT protein levels, we added an additional PLT decay term to the lowermost columella cells.

Sup Table 4: PLT parameters

Parameter	Description	Salvi2020 value	Current value	Dimension
p_{Plts}	*Max PLT production*	*0.00875*	*0.013125*	*[]*s⁻¹*
d_{Plts}	PLT degradation	0.0000175	0.0000175	s^{-1}
$KM_{D,Plts}$	DD Km half max production	60	60	[]
Pltsdiff	PLT diffusion across membrane	0.0000534	0.0000534	s^{-1}

CK signalling

Based on the experimental data from Salvi et al., 2020, we include the auxin dependent induction and PLT dependent repression of ARR12 and ARR1. In contrast to ARR12, ARR1 signalling becomes fully active only at 5dpg (Moubayidin et al., 2010). During early development ARR1 activity is repressed by giberellin, which levels decline over the course of development (Muraro et al., 2016; Moubayidin et al., 2010). We model this through a time dependent repressor for ARR1 (Eq 10, $timeREP_{ARR1}$), which remains fully active until 4.5 dpg, after which it gradually decreases in activity until it is completely gone at 5 dpg. This ensures that the full activation of ARR1 at 5dpg arises in gradual manner.

$$\frac{d\mathrm{ARR}_1}{dt} = \frac{p_{ARR1} * Auxin^2}{Auxin^2 + KM^2_{Auxin,ARR1}} * TimeRE \frac{P_{ARR1} * KM^2_{Plts,ARR1}}{\mathrm{Plts}^2 + \mathrm{KM}^2_{Plts,ARR1}} - d_{ARR1} * \mathrm{ARR1}_1 \tag{9}$$

$$TimeREP_{ARR1} = min\left(1{,}max\left(0{,}\frac{(tpg - ARR1esxpressionstart)}{(ARR1maximumeffect - ARR1expressionstart)}\right)\right)$$

, where p_{ARR1} is the maximum ARR1 production rate, $KM_{Auxin,ARR1}$ the concentration of auxin at which ARR1 production is half maximum, tpg is the time post germination (developmenntal time) in seconds, ARR1expressionstart is the time at which ARR1 expression starts (i.e. GA levels start declining), ARRmaximumeffect is the time at which ARR1 becomes fully active (i.e. GA levels

have reached a minimum level), $KM_{Plts,ARR1}$ is the PLT concentration for which ARR1 production is repressed to half its maximum and d_{ARR1} is the ARR1 degradation rate.

$$\frac{d\text{ARR}_{12}}{dt} = p_{ARR12}\frac{Auxin^2}{Auxin^2 + AuxKM_{ARR12}^2} + \frac{PltsKM_{ARR12}^2}{Plts^2 PltsKM_{ARR12}^2} - d_{ARR12} * ARR12 \quad (10)$$

, where p_{ARR12} is the maximum ARR12 production rate, $KM_{Auxin,ARR12}$ the concentration of auxin at which ARR12 production is half maximum $KM_{Plts,ARR12}$ is the PLT concentration for which ARR12 production is repressed to half its maximum and d_{ARR12} is the ARR12 degradation rate.

The ARR1 and ARR12 parameter values provided below are the default values, and are used in all simulations unless explicitly stated otherwise (see Supplementary Table 5 and Supplementary Table 6 for ARR1 and ARR12 parameters respectively). Alternative parameter settings used in certain simulations are discussed in the hypothetical scenario section. All parameters not mentioned in that section maintain their default values.

Sup Table 5: ARR1 Parameters

Parameter	Description	Salvi2020 value	Current value	Dimension
p_{ARR1}	Maximum ARR1 production rate	0.02	0.02	$[]s^{-1}$
d_{ARR1}	ARR1 degradation rate	0.0002	0.0002	s^{-1}
$KM_{Auxin,ARR1}$	Auxin level at which ARR1 production is induced to half maximum	160	160	[]
$KM_{Plts,ARR1}$	PLT level at which ARR1 production is repressed to half maximum	30	30	[]
ARR1expressionstart	*runtime when ARR1 starts being expressed*	*259200(3dpg)*	*388800 (4.5dpg)*	*s*
ARR1fullexpression	*Runtime from when ARR1 is fully active*	*388800(4.5dpg)*	*432000 (5dpg)*	*s*
ARR1maximumeffect	*Multiplication of ARR1 relative to ARR12 effect*	*2*	*3*	*dimensionless*

Sup Table 6: ARR12 parameters

Parameter	Description	Salvi2020 value	Current value	Dimension
p_{ARR12}	Maximum ARR12 production rate	0.02	0.02	$[]s^{-1}$
d_{ARR12}	ARR12 degradation rate	0.0002	0.0002	s^{-1}
$KM_{Auxin,ARR12}$	Auxin level at which ARR12 production is induced to half maximum	175	160	[]
$KM_{Plts,ARR12}$	PLT level at which ARR12 production is repressed to half maximum	30	30	[]
$KM_{ARR12,Plts}$	ARR12 level at which PLT production is reduced to half its maximum	25	25	[]
ARR12Pltsrepressfrac	Maximum fraction of PLT repression by ARR12	1.0	1.0	dimensionless

Auxin production

Auxin production is restricted to intra-cellular grid points. We model a basal level of auxin production, as well as additional auxin production mediated via PLT-induced YUCCA3 (Santuari et al., 2016), which catalyzes a rate limiting step in auxin metabolism. We model YUCCA3 expression as:

$$\frac{d\mathrm{YUC3}}{dt} = \frac{p_{YUC3} * Plts^2}{Plts^2 + PltsKM_{YUC3}^2} - d_{YUC3} * YUC3 \qquad (12)$$

,where p_{Yuc} is the maximum YUCCA3 production rate, $KM_{Plts,YUC3}$ is the PLT value for which YUCCA production is half maximum and d_{Yuc3} is the YUCCA3 degradation rate. For parameters see Supplementary Table 7.

Sup Table 7: YUCCA parameters

Parameter	Description	Salvi2020 value	Current value	Dimension
p_{YUC3}	Maximum YUCCA production rate s^{-1}	0.03	0.03	s^{-1}
d_{YUC3}	Yucca degradation rate s^{-1}	0.0003	0.0003	$[]s^{-1}$
$KM_{Plts,YUC3}$	*Yucca production PLT KM*	*175*	*200*	*[]*
Ap_{YUC}	Yucca based auxin production rate	0.0015	0.0015	[]

Auxin degradation:

Auxin degradation is limited to intra-cellular grid points. We model a basal level of auxin degradation as well as additional GH dependent auxin degradation. Two GH enzymes are incorporated in our model, GH3.3 that is repressed by PLT (Santuari et al., 2016) and the CK induced GH3.17 that is expressed in the lateral root cap and epidermis (Di Mambro et al., 2017) GH3.3 and GH3.17 expression is modeled as:

$$\frac{d\mathrm{GH33}}{dt} = \frac{p_{GH33} * KM^2_{Plts,GH33}}{Plts^2 + KM^2_{Plts,GH33}} - d_{GH33} * \mathrm{GH33} \tag{32}$$

$$\frac{d\mathrm{GH317}}{dt} = \frac{\mathrm{p_{GH317}} * ARRs_{eff}}{ARRs^2_{eff} + ARRsKM^2_{GH317}} - \mathrm{d_{GH317}} * \mathrm{GH317} \tag{43}$$

,with p_{GH33} and p_{GH317} as the maximum production rates of GH3.3 and GH3.17 respectively, d_{GH33} and d_{GH317} as the degradation rates of GH3.3 and GH3.17 respectively. $KM_{Plts,GH33}$ is the PLT concentration for which GH33 production is repressed to half its maximum. Finally $KM_{ARRs,GH317}$ is the combined ARR effect level for which GH317 production is at half maximum. For parameters see Supplementary Table 8.

Sup Table 8: GH parameters

Parameter	Description	Salvi2020 value	Current value	dimension
p_{GH33}	Maximum GH3.3 production rate s^{-1}	0.03	0.03	$[]s^{-1}$
d_{GH33}	GH3.3degradation rate	0.00003	0.00003	s^{-1}
$KM_{PltsGH33}$	PLT level at which GH3.3 production is half maximum	100 (Plts)	100 (Plts)	[]
Ad_{Gh33}	GH3.3 based auxin degradation per GH3.3(a.u.)	$1.45*10^{-7}$	$1.45*10^{-7}$	$[]^{-1}$

p_{GH317}	Maximum GH3.17 production rate	0.03	0.03	[]s^{-1}
d_{GH317}	GH3.17 degradation rate	0.0003	0.0003	s^{-1}
$KM_{ARRs,GH317}$	ARR level at which GH3.17 production is half maximum	75	130	[]
Ad_{GH317}	GH3.17 based auxin degradation per GH3.17(a.u.)	$1.16*10^{-6}$	$1.16*10^{-6}$	[]

Auxin import

We model a baseline passive auxin import , as well as active AUX/LAX importer mediated influx of auxin from the apoplast into the cell. Similar to our earlier work (van den Berg 2016), we incorporated the auxin dependent expression of AUX1 as follows:

$$\frac{dAUX1}{dt} = \frac{p_{AUX1} * Auxin^2}{Auxin^2 + \mathrm{KM}^2_{\mathrm{Auxin,AUX1}}} - d_{AUX1} * \mathrm{AUX1} \tag{54}$$

, where p_{AUX1} is the maximum AUX1 production rate and d_{AUX1} is the AUX1 degradation rate. $KM_{Auxin,AUX1}$ this the auxin level for which the AUX1 production is half maximum. For parameters see Supplementary Table 9.

Sup Table 9: AUX1 parameters

Parameter	Description	Salvi2020 value	Current value	Dimension
aux1fac	Aux1 influx strength	0.21	0.21	Dimensionless
p_{AUX1}	Aux1 maximum production	0.01	0.01	[]s^{-1}
$KM_{Auxin,AUX1}$	Aux1 production half saturation value for auxin	50	50	[]
decay_aux1	Aux1 degradation	0.0001	0.0001	[]s^{-1}

Auxin export

Auxin is trapped inside cells through protonation, making passive diffusion through the hydrophobic plasma membrane impossible. It thus only leaves cells through active export. A major determinant of active auxin export are the polarly localized PIN transporters. In our model we incorporate the dynamic regulation of PIN1, which has an endodermal and vascular expression pattern and predominantly basal orientation, and PIN2 which is expressed in the lateral root cap, and epidermis where it has a predominant apical orientation, and in the cortex where orientation switches from basal to apical as cells move from the meristem to the elongation zone (Figure 1A).

For both PIN1 and PIN2, we incorporate the SHY2 mediated repression downstream of ARR1 and ARR12. For this we take into account the previously reported stronger effect of ARR1 relative to ARR12 (Moubayidin et al., 2010). Additionally we incorporate the auxin dependent repression of SHY2 counteracting ARR-B mediated PIN repression. Finally, for PIN2 we incorporate its PLT dependent repression (Santuari et al., 2016). For simplicity, expression levels of other PIN types (PIN3, and PIN7 in the collumella) are kept constant.

$$\frac{dPIN1}{dt} = p_{PIN} * \mathrm{SHY2}_{reg} - d_{PIN} * \mathrm{PIN1}$$

$$\frac{dPIN2}{dt} = p_{PIN} * \mathrm{SHY2}_{\mathrm{reg}} * Plts_{reg} - d_{PIN} * \mathrm{PIN1}$$

$$SHY2_{reg} = 1 - \left(PIN_{ARRs} \frac{fac * KM^2_{ARRs,PINs}}{\mathrm{ARRs}^2_{eff} * \mathrm{KM}^2_{ARRs,PINs}}\right) * Auxin_{regSHY2}$$

$$\mathrm{ARRs}_{eff} = \mathrm{ARR1} * \mathrm{ARR1}maximumeffect + \mathrm{ARR12} \tag{65}$$

$$Auxin_{regSHY2} = 1 - \frac{Auxin^2}{Auxin^2 + AuxinKM^2_{SHY2}}$$

$$Plts_{reg} = \left(1 - PIN2_{Plts}fac + \frac{PIN2_{Plts}\mathrm{fac} * KM_{Plts,PIN2}}{Plts^2 * KM^2_{Plts,PIN2}}\right)$$

, where p_{PIN} is the maximum PIN production rate and d_{PIN} is the PIN degradation rate for both PIN1 and PIN2. $KM_{ARRs,PINs}$ is the combined ARR effect value for which the ARR PIN repression effect is half maximal, $KM_{Auxin,SHY2}$ is the auxin level at which the ARR effect is repressed to half its value and $KM_{Plts,PIN2}$ is the PLT value for which PIN2 is repressed to half of the remaining value. PIN_{ARRs}fac is the maximum factor by which PIN1 and PIN2 can be repressed by ARRs and $PIN2_{Plts}$fac is the maximum factor by which PLT can repress PIN2. For parameters see Supplementary Table 10.

Sup Table 10: Efflux parameters

Parameter	Description	Salvi2020 value	Current value	Dimension
pPUMP	Pin efflux strength	0.2	0.2	dimensionless
p_{PIN}	Pin maximum production	0.01	0.01	$[]s^{-1}$
d_{PIN}	Pin degradation	0.0001	0.0001	s^{-1}

Pin1regfac	Fraction pin 1 upregulation under PLT	0.0	0.0	0.0
$PIN2_{Plts}fac$	*Fraction pin2 downregulation under PLT*	*0.7*	*0.6*	*0.7*
$KM_{Plts,PIN2}$	Km of pin2 downregulation by PLT	80	80	[]
$PIN_{ARRs}fac$	Fraction pin downregulation under ARR(via SHY2)	0.3	0.3	dimensionless
$KM_{ARRs,PINs}$	Km of pin(s) downregulation by ARR	75	130	[]
$AuxinKM_{SHY2}$	Km of repression auxin repression of SHY2	225	225	[]

Combined Auxin dynamics

We model auxin dynamics on the grid level, enabling the simulation of intracellular and intra-apoplast auxin gradients. Changes in auxin levels arise from auxin production and degradation, import into and export from cells as well as diffusion inside cells and apoplast. For intra-cellular, membrane-localized grid points this results in the following differential equation:

$$\begin{aligned}\frac{\partial \text{Auxin}}{\partial t} &= Ap_b + Ap_{YUC} * YUC_3 - (Ad_b + Ad_{GH33} * \text{GH33} + Ad_{GH317} * GH317) * Auxin_{ij} \\ &+ \sum_{i,j} \left(i_{pas} + i_{act*AUX1_{ij}} * Auxin_{i\prime,j\prime} \right) - \sum_{i,j} \left(e_{pas} + e_{act*PIN_{ij}} * Auxin_{i,j} \right) \\ &+ D \left(\frac{\partial^2 \text{Auxin}}{\partial^2 x} + \frac{\partial^2 \text{Auxin}}{\partial^2 y} \right)\end{aligned} \quad (76)$$

, here Ap_b is a baseline, per grid point auxin production, which for QC, columella, and LRC cells is multiplied by a factor, 87.5 ,43.75 and 21, respectively. Ap_{YUC} is the YUCCA3 dependent auxin production in YUCCA3 expressing cells, Ad_b is the basal degradation rate, Ad_{GH33} is the GH3.3 mediated degradation and Ad_{GH317} is the GH3.17 mediated degradation in the epidermis and LRC.

At the interface between cell wall grid points and neighboring cellular grid points passive influx

as well as active AUX1 mediated import of auxin $i_{act*AUX1ij}$ takes place. Here, $AUX1_{ij}$ = $AUX1Prepat_{ij}$ * [AUX1] where AUX1Prepat represents the predefined cell and zonation type

dependent AUX1 expression pattern (Figure 3A), while[AUX1] represents the dynamically regulated concentration of AUX1(Eq 15) in the corresponding cell.

At this same interface auxin export also occurs. Active auxin export is mediated through several families of transporters, with a dominant role for the polarly localized PIN proteins (Wiśniewska et al., 2006).

In our model we consider both a constant active export mediated by ABCB transports e_{bas} as well as an active, PIN mediated export, $e_{act*PINij}$. Similar as for AUX1, here PIN_{ij} = $PINxPrepat_{ij}$ * [PINx]+$PIN2Prepat_{ij}$ * [PIN2] where. PINx1Prepat represents the predefined cell and zonation dependent PIN expression pattern of the relevant PIN type (Figure 1A) and [PINx] represents the dynamically regulated concentration of the relevant PIN type (Eq 16) and PIN2Prepat are predetermined according to cell file and zone as was the case in Salvi 2020. The combined PIN prepattern is shown in figure 1A. Prepatterns for individual PIN types can found in Salvi2020 in Figure 3A. It is further explained earlier in the *spatial layout* section. See Apolar PIN transport section for the details in the experiment without polar auxin patterning. Auxin production and degradation occur only in intracellular grid points, auxin import and export occurs only between pairs of adjoining cell wall and cell membrane grid points, and auxin diffusion is limited to occur between adjoining cell or adjoining wall grid points. For parameters see Supplementary Table 11.

Sup Table 11: Basic Auxin parameters

Parameter	Description	Salvi2020 value	value	Dimension
APb_b	Basal auxin production	0.003	0.003	$[]s^{-1}$
Inf	*Auxin level shoot influx*	*0,8*	*0.6*	*[]*
Ad_b	Basal degradation	0.000058	0.000058	$\mathbf{s^{-1}}$
DauxC	Auxin diffusion in cell	600	600	$\mu m^2 s^{-1}$
DauxW	Auxin diffusion in wall	40	40	$\mu m^2 s^{-1}$
eff	Passive auxin efflux	1	1	$\mu m s^{-1}$
InAux	Passive auxin influx	2.5	2.5	$\mu m s^{-1}$

Model alterations relative to Salvi et al., 2020

PLT refitting

Experimental data (Mahonen et al., 2014, Yamada et al., 2019, Salvi et al., 2020) show that the domain of PLT transcription is spatially constrained to the region surrounding the QC, while the PLT protein gradient is substantially more extended. We successfully fitted our model parameters to closely reproduce these observations in the Salvi et al., 2020 model, but observed here that when lowering meristematic division rates, thereby reducing the dilution of PLT proteins as well as its upstream factors, a less well spatially constrained PLT transcriptional domain arose. We therefore refitted a subset of parameter values to more robustly reproduce the spatially constrained PLT transcriptional domain under a broad range of division rates. To achieve this we increased the level of auxin that via ARF and intermediate transcription factors results in half-maximum activation of PLT expression, constraining transcription more to the high auxin regime, while compensating for the loss in PLT expression by increasing the maximum PLT expression level. Additionally, other parameters, such as the PLT half saturation value for YUCCA expression had to be refit to obtain similar YUCCA activation for somewhat altered PLT spatial expression. If a parameter value was changed between the Salvi et al. 2020 model and the model applied in this paper, the tables below will show both original and new value.

Auxin production and influx

Compared to the Salvi et al., 2020 shoot auxin influx rate was reduced from 0.8 to 0.6 (see Sup Table 11), while the multiplication factors to enhance local auxin production in the QC, columella and lateral root cap where enhanced from 50, 25, 12, respectively to 87.5, 43,75 and 21, respectively (see Sup Table11). Combined, this resulted in a reduced dependence of root tip auxin levels on external auxin supply from the shoot. This reduced dependence was necessary to prevent wildtype adult meristems from collapsing in absence of shoot auxin influx, which would be inconsistent with earlier findings (Grieneisen et al., 2007).

ARR1 and ARR12 refitting

Experimental data show that the effect of ARR1 mutation on final meristem size is larger than that of the effect of ARR12 mutation. To reproduce this difference in affecting meristem size, in addition to the KRP2 mediated repression of cell division by ARR1, we need to incorporate a stronger auxin antagonism for ARR1 than ARR12. However, in the Salvi et al., 2020 paper -in absence of decisive evidence supporting a mechanistic basis for the enhanced auxin antagonism of ARR1- we took a conservative approach, applying only a factor 2 difference in the ARR1 versus ARR12 auxin

antagonism. In this paper we performed a MEME analysis demonstrating that ARR1 (along with other ARR-B proteins that bind to DELLA) differs from from ARR12 precisely in the region in which DELLA is known to bind ARR1. This supports the hypothesis that ARR12 does not interact with DELLA and therefore its effectiveness can not be enhanced by it. We thus felt justified to take a less conservative approach, enhancing the difference in ARR1 and ARR12 mediated auxin antagonism to a factor 3 by enhancing ARR1 auxin antagonism strength. At the same time the effect of ARR1 on KRP2 and hence division was reduced (see Sup Table 1). These changes enabled us to generate an improved fit to the meristem size data from Moubayidin et al., 2010, which demonstrated that a predominant part of the ARR1 effect on meristem size can by explained from its SHY2 mediated auxin antagonism (see Moubaydin et al., 2010, Figure Q)

Extended SCN

The transition between slowly dividing SCN cells and TA cells has been slightly altered. In the Salvi et al., 2020 model we incorporated one row of SCN cells dividing at the slowest division duration of 20 hours, a second row of cells dividing every 15 hours, after which further TA cell divisions took 10 hours. Experimentally, substantially slower SCN division rates, and a larger number of cell positions from the QC undergoing slower than the most rapid TA cell divisions has been reported(Rahni and Birnbaum, 2019). To increase the realism of our model we here incorporated a total of 3 SCN positions undergoing slower division, with default durations of 20, 15.5 and 14.5 hours when moving away from the QC.

Experimental parameter variations

Altered division rates

To investigate the impact of division location and rate on PLT gradient patterning we varied division rates in either the TA zone or the SCN. For the TA, we take all cells starting from a distance of 5 cell rows from the QC to the boundary of the MZ. Division duration was varied from the default of cell cycle duration of 10 hours to values between 6 and 16 hours per division. Cells that have divided over 3 times but have not yet reached the 5th cell row undergo one of two scenarios. First, if the TA division time is lower than 10 hours, these intermediate cells have a division time of 10 hours, allowing for a gradual transition in cell cycle duration between the SCN and TA. If the TA divides more slowly than 10 hours, these intermediate divisions already adopt TA division speed (see Supplemental Figure 1)
For simulations where we altered the division duration for the SCN, we alter the rates for the first division (default 20 hours), second division (default 15.5 hours), and third division (default 14.5 hours) with the same factor. So if we e.g. raise the division time of the first division to 32 hours, that is a 32/20=1.6 factor increase, the second division will take 15.5*1.6 = 24.8 hours, while the third

will take 14.5*1.6=23.2 hours. The division times mentioned in figure 6 show the division time of the first SCN division. See Sup Tables 12 and 13 for the used SCN division time and TA division times in Figure 6.

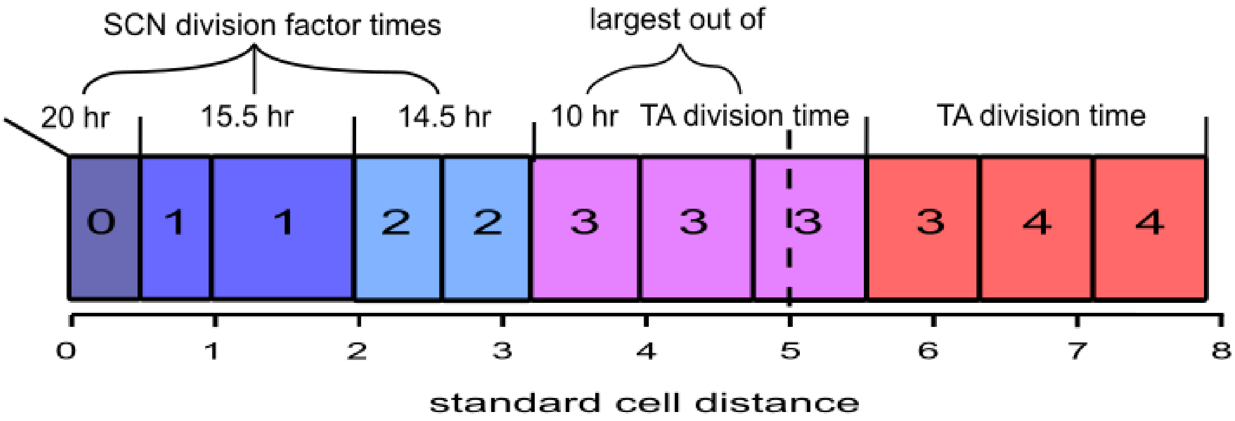

Supplemental Figure 1: Distribution of time to division in the SCN and TA for cells as they mature and undergo multiple divisions (number in cell). The blue cells division time are multiplied by the SCN division factor (see Sup Table 12 for the associated division times). Cells that have divided 3 times but have not yet passed the 5 cell standard distance from the start of the SCN divide either at once per 10 hours or at the TA division time (see Sup Table 13) if this is equal or larger than 10. Dividing cells beyond this always use the TA division time.

Sup Table 12: Range of durations for SCN divisions

First SCN division in hours	14	16	18	**20**	22	24	26	28	30	32
2nd division in hours	10.85	12..4	13.95	**15.5**	17.05	18.6	20.15	21.7	23.25	24.8
3rd division in hours	10.15	11.6	13.05	**14.5**	15.95	17.4	18.85	20.3	21.75	23.2

Sup Table 13: Range of durations for TA divisions

TA division in hours	6	8	**10**	12	14	16

No PLT dilution experiment

To investigate the importance of cell growth and division driven PLT dilution for meristem growth dynamics for a subset of simulations we eliminated the cell volume increase driven dilution specifically for the PLT protein. However, since this effectively produces PLT ex nihilo, we compensated for this by reduced YUCCA-mediated auxin production in order to more fairly demonstrate that absence of PLT dilution in itself is not incompatible with finite meristem size increase. YUCCA production was altered as follows (see Sup Table 14).

Sup Table 14: No dilution experiment altered YUCCA

Parameter	Default value	Alternative values:		
YUCCA prod<br>AbP_{YUC}	50*base<br>0.0015	5*base<br>0.00015	7.5*base<br>0.000225	8*base<br>0.00024

Apolar PIN transport

To investigate the importance of the polarity of PIN transport we constructed an alternative prepattern where a generic, apolarly localized PIN is expressed in all cells across all tissues. To maintain an approximately equal cellular auxin export rate we lower the membrane level of this apolar PIN to 30% of the level PINs normally have on their apical (PIN2) or basal (PIN1) membrane. To retain a little bit of polarity and ensure that the high auxin domain initiates at the root tip, we added to this generic, apolar PIN a small amount of basal PIN1, reduced to 3% of its normal basal level in the vascular tissues. (Effectively a 10% addition to the uniform PIN in the downwards direction). Finally, we turned off shoot auxin influx, as without sufficiently polar transport the thus supplied auxin would not reach the root tip and instead cause the high auxin domain to start at the shootward boundary. See Sup Table 15 for the parameters used, and Sup Table 16 for the different YUCCA production values used in Figure 2.

Sup Table 15: No Polar transport altered parameters

Parameter	Default value	Alternative values:
pPIN1	0.01	0.0003
pPIN2	0.01	0.003
Inf	0.6	0.0
p_{plts}	0.01325	0.13125
d_{plts}	0.0000175	0.000175
$p_{A,B,C,D}$	0.0065	0.065
$d_{A,B,C,D}$	0.000065	0.00065

Sup Table 16: No Polar transport YUCCA production levels

Parameter	Default value	Alternative values:						
YUCCA prod<br>Ab_{PYUC}	50<br>0.0015	70<br>0.0021	73<br>0.00219	73.75<br>0.0022125	73.875<br>0.00221625	74<br>0.00222	75<br>0.00225	100<br>0.003

Regional modifications in PLT cell-to-cell movement

In our model, a plasmodesmata aperture prepattern based on experimental data determines the rate of flux of PLT between two adjacent cells (see Supplemental Figure2A for the prepattern). Here we have maintained this pattern as the default plasmodesmatal aperture pattern, but for particular simulations also use alternative patterns specifically altering PLT flux rates around the SCN, or longitudinally in the TA(see Supplemental Figure 2B-D). For the TA, we only alter plasmodesmatal aperture in the

longitudinal direction. This enables us to argue that the observed reduction in PLT gradient length and meristem size occurring for increased intercellular PLT flux does not arise from an increased efflux of PLT to lateral tissues hampering longitudinal PLT spread. See Sup Table 17 for the parameters used in Figure A4 and Figure 5.

Sup Table 17: Altered diffusion rates

Parameter	Original value	Altered values					
Pltsdiff	0.0000534	0.00000534	0.00001068	0.0000267	0.0001068	0.000267	0.000534
Longitudinal TA diffusion rate	0.0000534	0.00000534	0.00001068	0.0000267	0.0001068	0.000267	0.000534
SCN diffusion rate	0.0000534	0.00000534	0.00001068	0.0000267	0.0001068	0.000267	0.000534

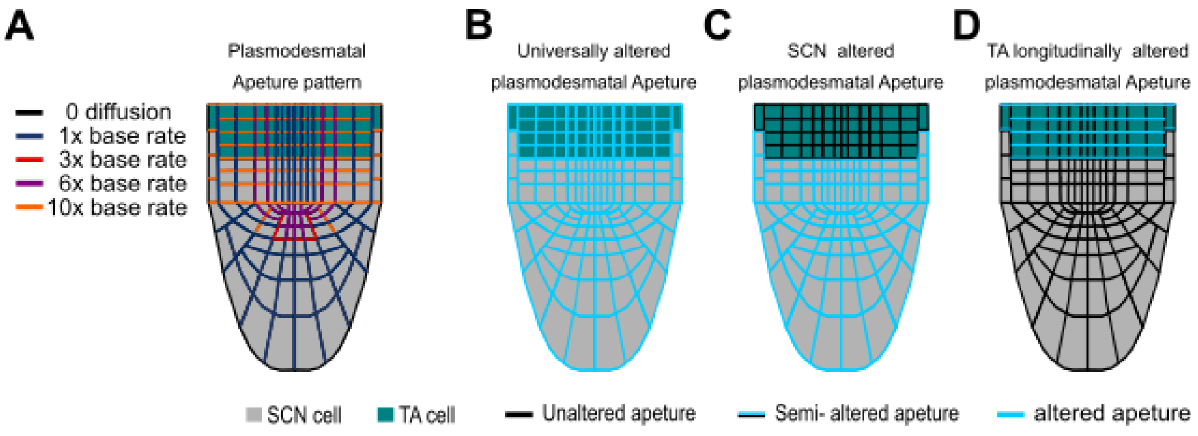

Supplemental Figure 2: Visualization of Plasmodesmata aperture in the root tip model. A) Standard Plasmodesmatal aperture. B-D) Altered plasmodesmatal aperture for the universally altered rate in blue, unaltered aperture in black. (B), SCN altered aperture (C), and longitudinally altered TA plasmodesmatal aperture (D).

Altered influx:

We reduce or increase auxin influx at the top of the simulated vasculature tissue to mimic altered auxin influx from the not explicitly simulated shoot (see Sup Table 18).

Sup Table 18: Altered shoot influx values

Parameter	Original value	Altered values									
Inf	0.6	0.0	0.1	0.2	0.3	0.4	0.5	0.7	0.8	0.9	1.0

Altered ARR1 and ARR12

In Figure 4C we demonstrate the difference between ARR1 and ARR12 in terms of temporal activity and dominance. The parameters used in this figure can be found in Sup Table19, along with

the altered timing and auxin influx levels of Figure 4D. (note that in practice expression of ARRs with 0 delay is delayed to 1.5dpg because of dominant PLT repression until then).

Sup Table 19: Altered ARR experiment parameters

Parameter	Original value	Altered values	
P_{ARR12}	0.02	0.01	0.04
P_{ARR1}	0.02	0.01	0.04
DelayARR1	4.5dpg	0dpg	
DelayARR12	0 dpg	4.5dpg	
Inf	0.6	0.55	

Single compartment model

The simplest of the three models applied in this paper is a single compartment model. This consists of two variables, Auxin and PLT, and is intended to capture in a simplified manner the essence of auxin PLT feedback dynamics. In the full root tip model, auxin levels depend on shoot auxin influx, a baseline production and degradation, a PLT-induced YUCCA mediated auxin production, a PLT-repressed GH3.3 mediated auxin degradation and an ARR-B induced GH3.17 mediated auxin degradation (based on equations 11 and 16). To simplify matters, in the single compartment model, baseline auxin production and shoot auxin influx are combined into a single influx term. Additionally, instead of incorporating both the PLT driven increase of auxin production and decrease of auxin degradation which have similar effects on steady state auxin levels, we restrict the modeled PLT dependence of auxin to the positive effect on auxin production.
Finally, since this model focuses on the auxin PLT feedback in absence of cytokinin signalling, we ignore the ARR-B mediated increase in auxin degradation. Combined this results in equation 17.

Reversely, PLT levels depend -with a certain time delay- on auxin levels via an ARF signalling dependent cascade of 4 transcription factors (Equations 7 and 8). In the single compartment model, for simplicity we ignore this time delay, modeling PLT expression as being directly dependent on ARF levels, resulting in equation 18:

$$\frac{d\text{Auxin}}{dt} = sizedep * Ap_{YUC} * YUC_3 - Ad_b * Auxin + influx \tag{17}$$

$$\frac{d\text{Plts}}{dt} = \frac{p_{Plts} * ARF_{free}^2}{{ARF_{free}^2}^2 + \text{KM}_{ARF,Plts}^2} - d_{Plts} * \text{Plts} \tag{18}$$

Note that in absence of a subdivision of this compartment into individual cells we also do not consider cellular auxin import or export. The model thus only considers local auxin production and degradation, and shoot auxin influx, but not intra compartment transport of auxin. Since auxin

production is size dependent in the full root tip model, we use Sizedep to scale our model auxin production to the cells average production level.

Combined this would result in a total of 4 ODEs, the 2 for auxin levels and PLT described in Eq. 17-18, but also 2 equations for free ARF levels (Eq 6), and PLT-dependent YUCCA expression (Eq11). To enable 2D phase plane analysis, we applied QSSAs for both ARF levels and YUCCA expression, further reducing the dimensionality of the single compartment model to 2 ODEs

The YUCCA QSSA can be easily derived from equation 11, and can then be inserted into the auxin equation:

$$\mathrm{YUC3} = \frac{\frac{p_{YUC3}}{d_{YUC3}} * Plts^2}{Plts^2 + PltsKM_{YUC3}^2}$$

$$\frac{d\mathrm{Auxin}}{dt} = sizedep * A \frac{p_{YUC} * \frac{P_{YUC}}{d_{YUC}} * Plts^2}{Plts^2 + \mathrm{KM}_{PLT,YUC}^2} - Ad_b * Auxin + influx \tag{19}$$

We can subsequently introduce a new auxin production parameter Paux, which we define as $Paux = A\frac{p_{YUC}*p_{YUC}}{d_{YUC}} * sizedep = \frac{\frac{0.0015*0.03}{0.0003}*3}{4} = 0.112$. This results in the auxin differential equation seen in equation 25.

An analytical QSS expression for ARF can not be easily obtained, therefore we choose to derive an approximate QSS expression. For this we first put DauxIAA basal to zero, based on the observation that it only provides a minor amount of extra transcription when auxin levels are very low, and becomes irrelevant when auxin >100 , which is still far below the level of auxin required for significant ARF activation (~5% with a km of 435, see Supplemental Figure 3). In the higher auxin regime we are interested in, the equation functionally thus becomes:

$$\mathrm{ARF}_{free} = \frac{diss * ARF_{total}}{\left(diss + \frac{ass * P_{AUX/IAA}}{d_{AUX/IAA<TIR1}^2 * \mathrm{Auxin}^2}\right)} \tag{20}$$

Which can be rewritten as:

$$\text{ARF}_{free} = \frac{diss * ARF_{total} * d^2_{AUX/IAA<TIR1} * \text{Auxin}^2}{\left(diss * d^2_{AUX/IAA<TIR1} * \text{Auxin}^2 + ass * P_{AUX/IAA}\right)}$$

$$\text{ARF}_{free} = \frac{ARF_{total} * \text{Auxin}^2}{\left(\text{Auxin}^2 + \frac{ass * P_{AUX/IAA}}{diss * d^2_{AUX/IAA<TIR1}}\right)} \tag{21}$$

$$\text{ARF}_{free} = \frac{ARF_{total} * \text{Auxin}^2}{\left(\text{Auxin}^2 + \left(\sqrt{\left(\frac{ass * P_{AUX/IAA}}{diss * d^2_{AUX/IAA<TIR1}}\right)}\right)^2\right)}$$

Where we can define a new parameter:

$$KM_{Auxin,ARF} = \sqrt{\frac{ass * P_{AUX/IAA}}{diss * d^2_{AUX/IAA<TIR1}}} = \sqrt{\frac{0.01 * 0.01}{0.001 * 0.000725^2}} = 436 \tag{22}$$

to simplify the writing of our equation

In the PLT ODE, ARF can then be substituted by this approximate ARF QSS expression resulting in:

$$\frac{d\text{Plts}}{dt} = \frac{p_{Plts} * \left(\frac{Auxin^2}{Auxin^2 + \text{KM}^2_{Auxin,ARF}}\right)^2}{\left(\frac{Auxin^2}{Auxin^2 + \text{KM}^2_{Auxin,ARF}}\right)^2 + KM^2_{ARF,Plts}} - d_{Plts} * \text{Plts} \tag{23}$$

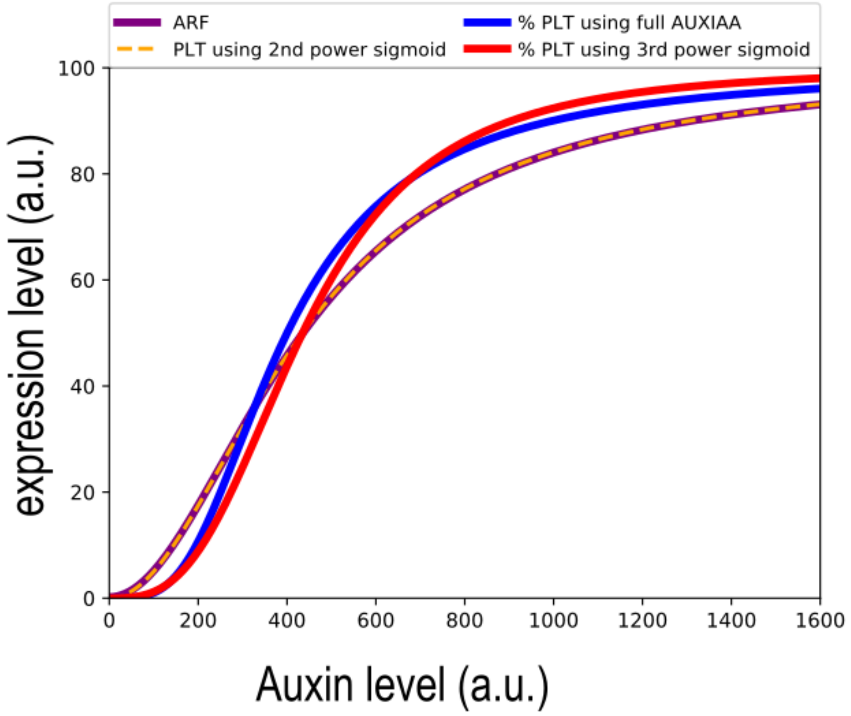

Supplemental Figure 3:Comparison between the root tip model sigmoids and the simplified sigmoid with a higher power used in the single and two component ODE models. Purple Line shows ARF steady state in full model. Orange dashed line shows that our equation

We further simplify this equation by replacing this complex sigmoid with a simpler higher power sigmoid:

$$\frac{dPlts}{dt} = \frac{p_{Plts} * Auxin^3}{Auxin^3 + \mathrm{KM}^3_{Auxin,Plts}} - d_{Plts} * Plts \tag{24}$$

See the blue and red lines in Figure 1A for a comparison between the two.

Taken together, the 2D Single-compartment model is thus given by:

$$\begin{aligned} \frac{d\mathrm{Auxin}}{dt} &= \frac{p_{Aux} * PLT^2}{Plts^2 + \mathrm{KM}^2_{PLT,Auxin}} - d_{Aux} * Auxin + influx \\ \frac{dPlts}{dt} &= \frac{p_{Plts} * Auxin^3}{Auxin^3 + \mathrm{KM}^3_{Auxin,Plts}} - d_{Plts} * Plts \end{aligned} \tag{25}$$

Sup Table 20: Single-compartment model additional parameters

Parameter	Value	Dimension
P_{Aux}	0.1125	$[]s^{-1}$
d_{Aux}	0.0001	s-1
$KM_{Plts,YUC}$	200	[]
Influx	0.0	$[]s^{-1}$
p_{Plts}	0.01312	$[]s^{-1}$
$KM_{Auxin,Plts}$	435	[]
d_{Plts}	0.0000175	s^{-1}

The YUCCA auxin production levels from Figure 1K and the Auxin shoot influx level from Figure 1I can be found in Sup Table 21.

Sup Table 21: Alternative single compartment settings

Parameter	Original value	Altered Value	
P_{Aux}	0.1125	0.045	0.0675
Influx	0.01	0.01	

Two-compartment model

The two compartment model is an extension of the above described single compartment model, generated through coupling two sets of the single compartment model equations together to model an SCN compartment and a TA compartment connected through auxin transport. To model the effect of cell growth and division, we incorporate a PLT protein level dilution term. Differences in division rates thus translate into differences in the size of this PLT dilution term. In addition we add auxin transport to the system, with PIN mediated transport between the two compartments of which the polarity/directionality can be varied, and shoot derived auxin influx into the TA compartment. Combined this results in the following system of equations:

$$\frac{dAuxin_{SCN}}{dt} = \frac{p_{Aux} * {Plts_{SCN}}^2}{{Plts_{SCN}}^2 + \mathrm{KM}^3_{PLT,YUC}}$$
$$-(d_{Aux} + Trans_{apolar}) * Auxin_{SCN} + (Trans_{apolar} + Trans_{polar}) * Auxin_{TA}$$
$$\frac{dAuxin_{TA}}{dt} = \frac{p_{Aux} * {Plts_{TA}}^2}{{Plts_{TA}}^2 + \mathrm{KM}^3_{Plts,YUC}} + influx$$
$$-(d_{Aux} + 2 * Trans_{apolar} + Trans_{polar}) * Auxin_{TA} + Trans_{apolar} * Auxin_{SCN} \quad (26)$$
$$\frac{dPlts_{SCN}}{dt} = \frac{p_{Plts} * Auxin^3_{SCN}}{Auxin^3_{SCN} + \mathrm{KM}^3_{Auxin,Plts}} - (d_{Plts} + dil_{SCN}) * Plts_{SCN}$$
$$\frac{dPlts_{TA}}{dt} = \frac{p_{Plts} * Auxin^3_{TA}}{Auxin^3_{TA} + \mathrm{KM}^3_{Auxin,Plts}} - (d_{Plts} + dil_{TA}) * Plts_{TA}$$

,where $Trans_{Apolar}$ is the baseline apolar auxin transport rate between cell compartments and $Trans_{Polar}$ is the rate at which auxin is rapidly transported from the TA to the SCN. Alternative parameters used in Figure 3F and Figure A2D,E are shown in Sup Table 23.

Sup Table 22 Two-compartment model parameters

Parameter	Value	Dimension
$Trans_{Apolar}$	0.00001	s^{-1}
$Trans_{Polar}$	0, or 0.01	s^{-1}
Influx	0	$[]s^{-1}$

Sup Table 23 Altered influx levels in Two-compartment model

Parameter	Original value	Altered values		
Influx	0	0.5	0.75	1.0
$Trans_{Apolar}$	0.00001	0.0001	0.001	

Model code and simulation

The models were written in C++. Numerical integration of cell-level ODEs was done using simple Euler forward integration, using a timestep of 2s. For integration of the PDE describing auxin dynamics, an alternating direction implicit integration scheme was used (Peaceman and Rachford, 1955), with a timestep of 0.2s and a spacestep of 1 micron.

The source code of the model is made publicly available and can be downloaded from http://bioinformatics.bio.uu.nl/khwjtuss/AuxinCytokinin/PlethoraRoot/SalviContinued

References

Band, L.R., Wells, D.M., Fozard, J.A., Ghetiu, T., French, A.P., Pound, M.P., Wilson, M.H., Yu, L., Li, W., Hijazi, H.I., et al. (2014). Systems analysis of auxin transport in the Arabidopsis root apex. Plant Cell *26*, 862–875.

van den Berg, T., Korver, R.A., Testerink, C., and ten Tusscher, K.H.W.J. (2016). Modeling halotropism: a key role for root tip architecture and reflux loop remodeling in redistributing auxin. Development *143*, 3350–3362.

Boudonck, K., Dolan, L., and Shaw, P.J. (1998). Coiled body numbers in the Arabidopsis root epidermis are regulated by cell type, developmental stage and cell cycle parameters. Journal of Cell Science *111*, 3687–3694.

Di Mambro, R., De Ruvo, M., Pacifici, E., Salvi, E., Sozzani, R., Benfey, P.N., Busch, W., Novak, O., Ljung, K., Di Paola, L., et al. (2017). Auxin minimum triggers the developmental switch from cell division to cell differentiation in the Arabidopsis root. Proc. Natl. Acad. Sci. U.S.A. *114*, E7641–E7649.

Farcot, E., Lavedrine, C., and Vernoux, T. (2015). A Modular Analysis of the Auxin Signalling Network. PLOS ONE *10*, e0122231.

Grieneisen, V.A., Xu, J., Marée, A.F.M., Hogeweg, P., and Scheres, B. (2007). Auxin transport is sufficient to generate a maximum and gradient guiding root growth. Nature *449*, 1008–1013.

Guilfoyle, T.J. (2015). The PB1 Domain in Auxin Response Factor and Aux/IAA Proteins: A Versatile Protein Interaction Module in the Auxin Response[OPEN]. Plant Cell *27*, 33–43.

Kim, J., Harter, K., and Theologis, A. (1997). Protein–protein interactions among the Aux/IAA proteins. PNAS *94*, 11786–11791.

Korasick, D.A., Westfall, C.S., Lee, S.G., Nanao, M.H., Dumas, R., Hagen, G., Guilfoyle, T.J., Jez, J.M., and Strader, L.C. (2014). Molecular basis for AUXIN RESPONSE FACTOR protein interaction and the control of auxin response repression. PNAS *111*, 5427–5432.

Krichevsky, A., Zaltsman, A., Kozlovsky, S.V., Tian, G.-W., and Citovsky, V. (2009). Regulation of root elongation by histone acetylation in Arabidopsis. J. Mol. Biol. *385*, 45–50.

Ljung, K., Hull, A.K., Celenza, J., Yamada, M., Estelle, M., Normanly, J., and Sandberg, G. (2005). Sites and Regulation of Auxin Biosynthesis in Arabidopsis Roots. The Plant Cell *17*, 1090–1104.

Mähönen, A.P., Tusscher, K. ten, Siligato, R., Smetana, O., Díaz-Triviño, S., Salojärvi, J., Wachsman, G., Prasad, K., Heidstra, R., and Scheres, B. (2014). PLETHORA gradient formation mechanism separates auxin responses. Nature *515*, 125–129.

Moubayidin, L., Perilli, S., Dello Ioio, R., Di Mambro, R., Costantino, P., and Sabatini, S. (2010). The Rate of Cell Differentiation Controls the Arabidopsis Root Meristem Growth Phase. Current Biology *20*, 1138–1143.

Muraro, D., Larrieu, A., Lucas, M., Chopard, J., Byrne, H., Godin, C., and King, J. (2016). A multi-scale model of the interplay between cell signalling and hormone transport in specifying the root meristem of Arabidopsis thaliana. Journal of Theoretical Biology *404*, 182–205.

Pacifici, E., Mambro, R.D., Ioio, R.D., Costantino, P., and Sabatini, S. (2018). Acidic cell elongation drives cell differentiation in the Arabidopsis root. The EMBO Journal *37*, e99134.

Peaceman, D.W., and Rachford, H.H. (1955). The Numerical Solution of Parabolic and Elliptic Differential Equations. Journal of the Society for Industrial and Applied Mathematics *3*, 28–41.

Rahni, R., and Birnbaum, K.D. (2019). Week-long imaging of cell divisions in the Arabidopsis root meristem. Plant Methods *15*, 30.

Rouse, D., Mackay, P., Stirnberg, P., Estelle, M., and Leyser, O. (1998). Changes in auxin response from mutations in an AUX/IAA gene. Science *279*, 1371–1373.

Santuari, L., Sanchez-Perez, G.F., Luijten, M., Rutjens, B., Terpstra, I., Berke, L., Gorte, M., Prasad, K., Bao, D., Timmermans-Hereijgers, J.L.P.M., et al. (2016). The PLETHORA Gene Regulatory Network Guides Growth and Cell Differentiation in Arabidopsis Roots. The Plant Cell Online *28*, 2937–2951.

Scacchi, E., Salinas, P., Gujas, B., Santuari, L., Krogan, N., Ragni, L., Berleth, T., and Hardtke, C.S. (2010). Spatio-temporal sequence of cross-regulatory events in root meristem growth. PNAS *107*, 22734–22739.

Stepanova, A.N., Robertson-Hoyt, J., Yun, J., Benavente, L.M., Xie, D.-Y., Doležal, K., Schlereth, A., Jürgens, G., and Alonso, J.M. (2008). TAA1-Mediated Auxin Biosynthesis Is Essential for Hormone Crosstalk and Plant Development. Cell *133*, 177–191.

Takatsuka, H., Higaki, T., and Umeda, M. (2018). Actin Reorganization Triggers Rapid Cell Elongation in Roots. Plant Physiology *178*, 1130–1141.

Verbelen, J.-P., De Cnodder, T., Le, J., Vissenberg, K., and Baluška, F. (2006). The Root Apex of Arabidopsis thaliana Consists of Four Distinct Zones of Growth Activities. Plant Signal Behav *1*, 296–304.

Wendrich, J.R., Möller, B.K., Li, S., Saiga, S., Sozzani, R., Benfey, P.N., Rybel, B.D., and Weijers, D. (2017). Framework for gradual progression of cell ontogeny in the Arabidopsis root meristem. PNAS *114*, E8922–E8929.
